# Association between preoperative persistent hyperglycemia and postoperative delirium in geriatric hip fracture patients

**DOI:** 10.1186/s12877-024-05192-x

**Published:** 2024-07-08

**Authors:** Wei Wang, Yingqi Zhang, Wei Yao, Wanyun Tang, Yuhao Li, Hongbo Sun, Wenbo Ding

**Affiliations:** 1 Department of Orthopedics, Dandong Central Hospital, China Medical University, Dandong, China; 2 School of Clinical Medicine, Dalian Medical University, Dalian, China; 3 Dandong Central Hospital, China Medical University, No. 338 Jinshan Street, Zhenxing District, Dandong, Liaoning Province 118002 P.R. China

**Keywords:** Hip fracture, Glucose levels, Hyperglycemia, Postoperative delirium, POD

## Abstract

**Background:**

The management of preoperative blood glucose levels in reducing the incidence of postoperative delirium (POD) remains controversial. This study aims to investigate the impact of preoperative persistent hyperglycemia on POD in geriatric patients with hip fractures.

**Methods:**

This retrospective cohort study analyzed medical records of patients who underwent hip fracture surgery at a tertiary medical institution between January 2013 and November 2023. Patients were categorized based on preoperative hyperglycemia (hyperglycemia defined as ≥ 6.1mmol/L), clinical classification of hyperglycemia, and percentile thresholds. Multivariate logistic regression and propensity score matching analysis (PSM) were employed to assess the association between different levels of preoperative glucose and POD. Subgroup analysis was conducted to explore potential interactions.

**Results:**

A total of 1440 patients were included in this study, with an incidence rate of POD at 19.1% (275/1440). Utilizing multiple logistic analysis, we found that patients with hyperglycemia had a 1.65-fold increased risk of experiencing POD compared to those with normal preoperative glucose levels (95% CI: 1.17–2.32). Moreover, a significant upward trend was discerned in both the strength of association and the predicted probability of POD with higher preoperative glucose levels. PSM did not alter this trend, even after meticulous adjustments for potential confounding factors. Additionally, when treating preoperative glucose levels as a continuous variable, we observed a 6% increase in the risk of POD (95% CI: 1-12%) with each 1mmol/L elevation in preoperative glucose levels.

**Conclusions:**

There exists a clear linear dose-response relationship between preoperative blood glucose levels and the risk of POD. Higher preoperative hyperglycemia was associated with a greater risk of POD.

**Clinical trial number:**

NCT06473324.

**Supplementary Information:**

The online version contains supplementary material available at 10.1186/s12877-024-05192-x.

## Introduction

With the global aging population on the rise, there is a corresponding increase in the number of geriatric individuals requiring surgical treatment for hip fractures. It is projected that by 2050, there will be approximately 6 million cases of hip fractures worldwide [1, 2]. In the United States alone, around 300,000 geriatric patients suffer from hip fractures each year [3]. Unfortunately, POD has emerged as a common complication following surgery for geriatric hip fractures, with an incidence rate ranging from 4–53% [4–6]. Numerous studies have demonstrated that POD not only escalates medical costs and hospitalization duration but also raises mortality rates at six months and one year [7–9]. In severe cases, it can even result in permanent cognitive impairment [10, 11].

POD manifests as acute cognitive dysfunction and consciousness disorder, characterized by changes in attention or consciousness that cannot be attributed to pre-existing cognitive impairments [12, 13]. Typical symptoms include hallucinations, delusions, agitated behavior, disordered speech, decreased attention, confusion, unclear consciousness, and disrupted sleep-wake cycles [10, 14]. Moreover, POD typically occurs within the first five days after surgery, particularly within the initial 48 h, and exhibits significant fluctuations [9, 14].

Research indicates that reduced brain energy metabolism and decreased brain glucose uptake may serve as potential pathogenic mechanisms for POD. Titlestad et al. [15]. conducted a study on the distribution differences of metabolites related to energy metabolism in the blood serum and cerebrospinal fluid of hip fracture patients with POD. The results revealed that when delirium occurred in hip fracture patients, there was a shift in brain glucose utilization towards ketone body metabolism. However, different studies yield varying conclusions regarding the impact of preoperative serum glucose levels on POD. For instance, Kotfis [16] and Lin [17] suggested that higher levels of glycosylated hemoglobin (HbA1c) and poor blood glucose control prior to surgery increase the risk of POD after cardiovascular surgery, regardless of the diabetes diagnosis. Zhang et al.‘s study [18] on spinal surgery demonstrated that elevated levels of glycosylated hemoglobin are independent risk factors for POD. Heymann’s research [19] indicated significantly higher blood glucose levels in POD patients in the intensive care unit (ICU) compared to non-delirium patients, suggesting a link between hyperactive delirium and elevated blood glucose levels.

However, Kris et al.‘s study [20, 21] emphasized that hypoglycemia may be linked to the development of POD in the ICU. Saager et al. [22]. reported that intraoperative blood glucose control (6.6 ± 1.0 mmol/L) resulted in a higher incidence of delirium in cardiac surgery patients compared to standard treatment (9.5 ± 1.6 mmol/L). Liu et al.‘s study [23] found that higher preoperative fasting blood glucose levels serve as a protective factor against POD in hip fracture patients. The conflicting findings regarding the impact of preoperative blood glucose levels on POD in hip fracture patients have created confusion in clinical management.

Therefore, clarification is needed to help guide preoperative glycemic management in order to decrease the risk of POD. Our study focuses on exploring the relationship between preoperative hyperglycemia and POD, rather than diabetes diagnosis. The aim of this study is to provide the following insights for clinical practice through a large-scale retrospective cohort study:

1. To furnish clinicians with accessible, promptly measurable, and dynamically monitorable biomarkers for POD, facilitating its prevention through the management of preoperative blood glucose levels.
2. To delineate the dose-response correlation between preoperative blood glucose levels and POD, ascertain the risk threshold of preoperative blood glucose levels, and scrutinize potential interactions with other risk factors, particularly within diabetic cohorts.

## Methods

### Study design and data collection

This retrospective cohort study aimed to collect electronic medical record data from our hospital spanning the period from January 2013 to November 2023. The data collection process was independently carried out by two authors (WW and WY), who diligently examined any discrepancies to ensure the accuracy of the data. Throughout the research endeavor, we adhered to the ethical principles outlined in the 1964 Helsinki Declaration and obtained approval from the Institutional Review Board (IRB) for all aspects of the study. In accordance with the IRB regulations, written informed consent was waived as the data collected did not contain personal information or compromise privacy.

### Patient selection

**Fig. 1:**
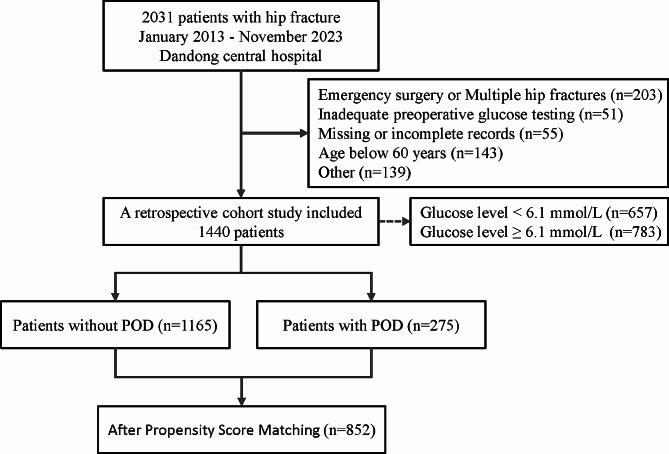
Flow diagram for selection of cohorts

The study included patients who had undergone surgical treatment for hip fractures, while excluding those who met the following criteria: (1) Multiple or pathological hip fractures; (2) Emergent surgery; (3) Age below 60 years; (4) Incomplete electronic medical records that were inaccessible or Preoperative blood glucose levels were assessed fewer than two times; (5) Presence of neurological and psychiatric disorders, except dementia. Please refer to Fig. 1 for a detailed screening process.

### Exposure

Upon admission and within 48 h before surgery, our hospital routinely conducts laboratory tests, including a complete blood cell count and biochemistry. The collection, processing, and analysis of blood samples follow the standard protocols of international biochemical laboratories. It is noteworthy that nurses collect the samples at the patient’s bedside every morning at 7 a.m. while the patient is fasting. For patients with poor blood sugar control upon admission, additional fasting blood glucose tests are performed based on the clinical need for blood sugar management. Similarly, nurses carry out these orders during morning blood collection. Persistent hyperglycemia is defined as having mild, moderate, or severe high blood glucose levels for two consecutive or more occasions, according to the research design referenced from other literature [24]. If the blood glucose levels on two or more occasions fall into different classifications of hyperglycemia, the lowest level of hyperglycemia will be documented, and the lowest blood glucose level will be recorded. For instance, if a patient’s blood glucose level is classified as mild hyperglycemia in one test and moderate hyperglycemia in another test, the patient will be ultimately classified as having persistent mild hyperglycemia.

The 2023 Clinical Practice Guidelines by the American Diabetes Association define normal blood glucose, as well as mild, moderate, and severe hyperglycemia, as serum glucose levels of <6.1mmol/L, 6.1-7.8mmol/L, 7.8-10.0mmol/L, and ≥ 10.0mmol/L, respectively [25, 26]. The patients’ preoperative blood glucose levels are grouped according to quartiles: the first quartile (Q1: 1.60-5.39mmol/L), the second quartile (Q2: 5.40-6.19mmol/L), the third quartile (Q3: 6.20-7.59mmol/L), and the fourth quartile (Q4: 7.60-35.9mmol/L).

### Outcome

The determination of POD primarily relies on the daily progress notes provided by the attending physician and the post-operative visit records documented by the anesthesiologist. Within the first 24 h after surgery, the anesthesiologist performs consciousness assessments at the patient’s bedside every 8 h. If the patient exhibits symptoms suggestive of POD, the attending physician will request a bedside consultation with an experienced psychiatrist. The diagnostic results and treatment recommendations will be documented by the attending physician. The diagnosis of delirium follows the criteria outlined in the Diagnostic and Statistical Manual of Mental Disorders, Fifth Edition (DSM-5), developed by the American Psychiatric Association in 2013. The Confusion Assessment Method (CAM) is commonly utilized as a diagnostic tool. The CAM incorporates the following criteria: (1) acute onset with a fluctuating course, (2) inattention, (3) disorganized thinking, and (4) altered level of consciousness. To receive a diagnosis of delirium, the patient must meet both criterion 1 and criterion 2, as well as either criterion 3 or criterion 4.

### Covariables

Drawing from the risk factors identified in prior research, we systematically gathered covariates from medical records and categorized them into four distinct groups: demographic variables, comorbidity variables, operative-related variables, and preoperative laboratory examination variables. Demographic variables encompassed age, gender, body mass index (BMI), smoking, and alcohol. Comorbidities included American Society of Anesthesiologists (ASA) classification, dementia, diabetes, hypertension, hypoglycemic medications, cardiovascular disease, and cerebrovascular disease. Surgery-related variables involved fracture type, surgical method, time to surgery, surgery duration, blood loss, and blood transfusion. Preoperative laboratory examinations comprised red blood cell count, neutrophil count, lymphocyte count, hemoglobin, serum albumin level, and total serum protein level.

### Statistical analysis

We utilized mean ± standard deviation or numbers (percentages) to delineate patients’ baseline characteristics. Categorical data were compared using a chi-square test, while continuous data were evaluated using a Kruskal-Wallis test. Multiple logistic regression analysis was employed to compute adjusted odds ratios (ORs) and 95% confidence intervals (CIs) by integrating covariates that exhibited statistical significance in univariate logistic regression analyses into subsequent multivariate logistic regression models.

To mitigate bias, we conducted PSM for all included covariates. In a 1:1 PSM approach, we adopted a nearest neighbor matching algorithm to align the normal blood glucose group with the persistent hyperglycemia group, with a standard deviation of 0.1 for the chi-square value. The standardized mean difference (SMD) was employed to detect potential imbalances between the two groups, with SMD ≥ 0.10 indicating evidence of disparity. Subsequently, logistic regression analysis was performed using the PSM-matched data to derive adjusted ORs and 95% CIs.

We further conducted subgroup analysis to explore potential interactions among variables. Patients were stratified into subgroups based on covariates, and independent logistic regression analyses were carried out for each subgroup. By comparing the disparity in ORs across different subgroups, we assessed the presence of interaction.

Statistical significance was established as a two-sided *p*-value < 0.05. All statistical procedures were executed using SPSS Statistics 25.0 for Windows (IBM Corp., Armonk, NY) and R software 4.3.1 for Windows (R Foundation for Statistical Computing, Boston, MA, USA).

## Results

From January 2013 to November 2023, a total of 2,031 electronic medical records were gathered. Following stringent inclusion and exclusion criteria, a retrospective cohort study was conducted on 1,440 patients. Among them, 783 patients (54.4%) exhibited preoperative persistent hyperglycemia (serum glucose levels ≥ 6.1 mmol/L on multiple occasions), while 275 patients (19.1%) experienced POD. The detailed selection process is depicted in Fig. 1.

**Fig. 2:**
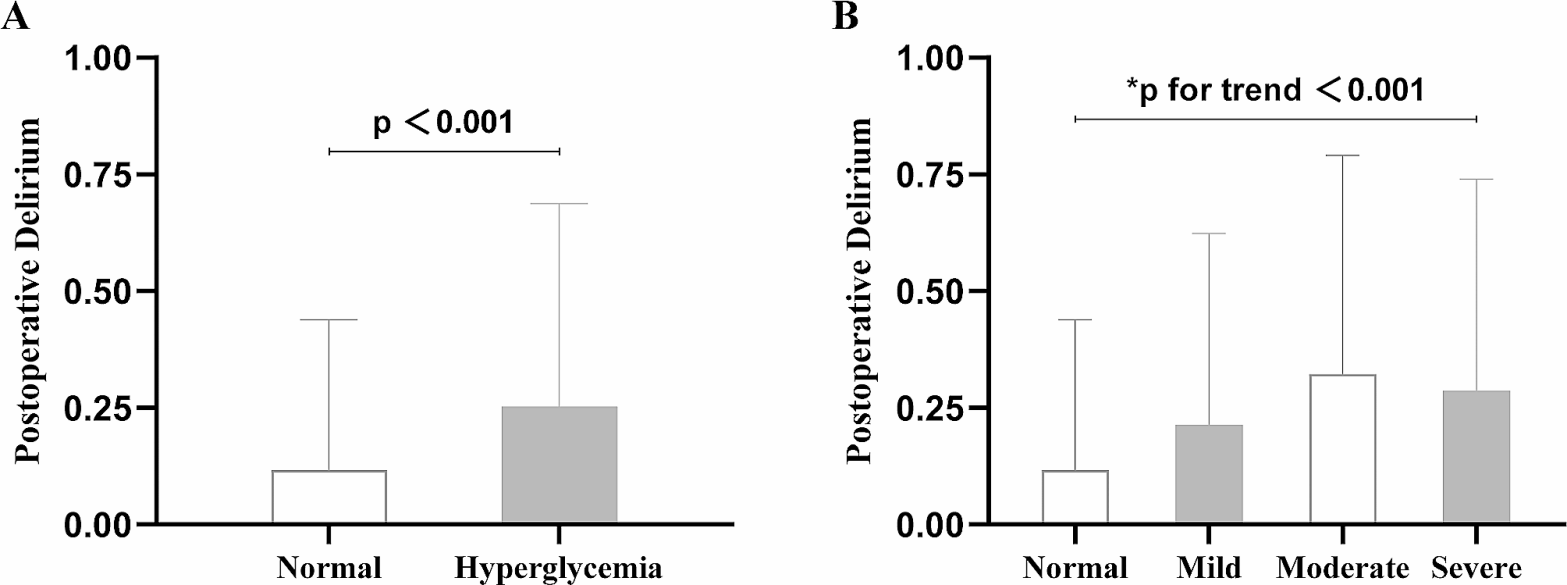
The column chart illustrates the incidence of POD in relation to varying preoperative blood glucose levels. In **panel A**, the incidence of POD was 11.7% in the normal preoperative glucose level group and 25.3% in the hyperglycemia group. **Panel B** demonstrates the variability in the incidence of POD across varying clinical thresholds of glucose levels: 11.7% in the normal level group, 21.3% in the mild hyperglycemia group, 32.2% in the moderate hyperglycemia group, and 28.7% in the severe hyperglycemia group

Table 1 presents the fundamental characteristics of patients categorized based on the clinical severity of preoperative hyperglycemia. The average age of the included geriatric hip fracture patients was 74.7 ± 9.8 years, with males comprising 39.7% of the sample. Obese patients (BMI ≥ 30.0 kg/m²) accounted for 20.3% of the total. Preoperative dementia was present in 6.0% of patients, hypertension in 51.0%, diabetes in 24.5%, cardiovascular disease in 30.9%, and cerebrovascular disease in 31.0%. Approximately 56.5% of patients had an ASA classification grade 3 or higher. Additionally, the incidence of POD was higher in the persistent hyperglycemia group compared to the normal blood glucose group (Fig. 2A, *p* < 0.001). Moreover, the incidence rates of POD for all clinical classifications of persistent hyperglycemia were higher than those in the normal blood glucose group (Fig. 2B, trend *p* < 0.001).

**Table 1:** Baseline characteristics of the patients based on preoperative glucose levels (mmol/L)

Characteristics	Total patients (*n* = 1440)	Clinical Classification of Glucose levels (mmol/L)				*p*
		Normal glucose level (<6.1, *n* = 657)	Mild Hyperglycemia (6.1–7.8, *n* = 450)	Moderate Hyperglycemia (7.8–10.0, *n* = 183)	Severe Hyperglycemia (≥ 10.0, *n* = 150)	
**Demographics**						
Age, years	74.7 ± 9.8	72.8 ± 10.3	76.2 ± 9.3	76.9 ± 9.1	76.0 ± 8.2	< 0.001
Male	571 (39.7%)	281 (42.8%)	177 (39.3%)	66 (36.1%)	47 (31.3%)	0.046
BMI ≥ 30.0 kg/m²	293 (20.3%)	104 (15.8%)	97 (21.6%)	48 (26.2%)	44 (29.3%)	< 0.001
Smoking	242 (16.8%)	122 (18.6%)	65 (14.4%)	38 (20.8%)	17 (11.3%)	0.036
Alcohol	166 (11.5%)	79 (12.0%)	51 (11.3%)	26 (14.2%)	10 (6.7%)	0.176
**Comorbidities**						
ASA classes ≥ III	814 (56.5%)	322 (49.0%)	268 (59.6%)	123 (67.2%)	101 (67.3%)	< 0.001
Dementia	86 (6.0%)	24 (3.7%)	36 (8.0%)	19 (10.4%)	7 (4.7%)	< 0.001
Hypertension	735 (51.0%)	272 (41.4%)	260 (57.8%)	116 (63.4%)	87 (58.0%)	< 0.001
Diabetes	353 (24.5%)	58 (8.8%)	100 (22.2%)	100 (54.6%)	95 (63.3%)	< 0.001
Hypoglycemic medications						< 0.001
Insulin	160 (11.1%)	30 (4.6%)	49 (10.9%)	54 (29.5%)	27 (18.0%)	
Oral hypoglycemic agents	180 (12.5%)	28 (4.3%)	51 (11.3%)	46 (25.1%)	55 (36.7%)	
Non-antidiabetic medications	1100 (76.4%)	599 (91.2%)	350 (77.8%)	83 (45.4%)	68 (45.3%)	
Cardiovascular diseases	445 (30.9%)	175 (26.6%)	144 (32.0%)	76 (41.5%)	50 (33.3%)	0.001
Cerebrovascular diseases	446 (31.0%)	173 (26.3%)	142 (31.6%)	74 (40.4%)	57 (38.0%)	< 0.001
**Operative-related Factors**						
Type of fracture						< 0.001
Femoral neck fracture	766 (53.2%)	404 (61.5%)	207 (46.0%)	81 (44.3%)	74 (49.3%)	
Intertrochanteric fracture	583 (40.5%)	220 (33.5%)	215 (47.8%)	83 (45.4%)	65 (43.3%)	
Subtrochanteric fracture	91 (6.3%)	33 (5.0%)	28 (6.2%)	19 (10.4%)	11 (7.3%)	
Type of surgery						< 0.001
Total Hip Arthroplasty	200 (13.9%)	105 (16.0%)	49 (10.9%)	21 (11.5%)	25 (16.7%)	
Hemiarthroplasty	356 (24.7%)	156 (23.7%)	116 (25.8%)	47 (25.7%)	37 (24.7%)	
Intramedullary nail fixation	480 (33.3%)	175 (26.6%)	172 (38.2%)	76 (41.5%)	57 (38.0%)	
Fixation with steel plate	177 (12.3%)	65 (9.9%)	69 (15.3%)	24 (13.1%)	19 (12.7%)	
Fixation with hollow nails	227 (15.8%)	156 (23.7%)	44 (9.8%)	15 (8.2%)	12 (8.0%)	
Time to surgery, days	5.5 ± 2.6	5.1 ± 2.6	5.6 ± 2.6	5.7 ± 2.7	6.3 ± 2.8	< 0.001
Duration of surgery, hours	1.7 ± 0.8	1.6 ± 0.7	1.7 ± 0.9	1.7 ± 0.8	1.7 ± 0.8	0.039
Operative blood loss, ml	172.9 ± 156.3	160.5 ± 148.7	184.7 ± 163.1	185.7 ± 165.8	176.4 ± 153.2	0.001
Blood transfusion	243 (16.9%)	85 (12.9%)	94 (20.9%)	41 (22.4%)	23 (15.3%)	< 0.001
**Preoperative Laboratory Tests**						
RBC count, ×10^9^/L	3.9 ± 0.7	4.0 ± 0.7	3.9 ± 0.7	3.8 ± 0.6	3.9 ± 0.7	< 0.001
NEU count, ×10^9^/L	6.8 ± 2.8	6.1 ± 2.5	7.2 ± 2.8	7.2 ± 2.7	7.7 ± 3.0	< 0.001
LYM count, ×10^9^/L	1.3 ± 0.7	1.4 ± 0.7	1.3 ± 0.7	1.2 ± 0.5	1.2 ± 0.5	< 0.001
HGB, g/L	119.7 ± 20.6	121.6 ± 20.4	118.1 ± 21.6	116.9 ± 18.3	120.1 ± 20.9	0.007
Albumin, g/L	37.6 ± 5.2	38.2 ± 5.0	37.3 ± 5.1	36.3 ± 5.8	37.4 ± 4.8	< 0.001
Total protein, g/L	65.0 ± 7.4	65.3 ± 6.9	64.8 ± 7.5	63.8 ± 8.6	65.4 ± 7.8	0.261

Continuous variables are presented as mean ± standard deviation, while categorical variables are represented by numbers (percentages)

BMI: Body Mass Index; ASA: American Society of Anesthesiologists; RBC: Red Blood Cells; NEU: Neutrophils; LYM: Lymphocytes; HGB: Hemoglobin

Multivariable logistic regression analysis, adjusting for covariates (refer to Supplementary e-Table 1), yielded the results presented in Table 2. Treating preoperative serum glucose levels as a continuous variable, the association between preoperative glucose levels and POD exhibited an OR of 1.06 (95% CI: 1.01–1.12, *p* = 0.016). In other words, for every 1 mmol/L increase in preoperative glucose level, the risk of POD increased by 6%. When treated as a binary classification, the OR value for the persistent hyperglycemia group, compared to the normal blood glucose group, was 1.65 (95% CI: 1.17–2.32, *p* = 0.004), indicating that preoperative persistent hyperglycemia can be considered an independent risk factor for POD. Further analysis demonstrated that, compared to the normal blood glucose group, the OR values for the mild, moderate, and severe persistent hyperglycemia groups were 1.50 (95% CI: 1.03–2.18), 1.84 (95% CI: 1.14–2.98), and 2.08 (95% CI: 1.24–3.50), respectively, with a trend *p*-value of 0.002. Similarly, compared to the first quartile, the OR values for the second, third, and fourth quartiles were 1.12 (95% CI: 0.68–1.85), 1.35 (95% CI: 0.84–2.19), and 1.84 (95% CI: 1.13-3.00), respectively. Therefore, a notable upward trend was observed in the correlation between preoperative persistent hyperglycemia and POD (trend *p* = 0.002 and *p* = 0.008) Additionally, to minimize confounding factors, PSM analysis was conducted in our study. The baseline characteristics of patients before and after matching are presented in Supplementary e-Table 2. Logistic regression analysis of the matched cohort revealed a gradual increase in the risk of POD with increasing preoperative glucose levels (trend *p* < 0.001), as detailed in Table 2.

**Table 2:** Unadjusted and adjusted association between preoperative glucose levels and postoperative delirium

Glucose (mmol/L)		Events, *n* (%)	Unadjusted OR (95% CI)	*P*	Multivariable regression adjusted OR (95% CI)	*P*	PSM adjusted OR (95% CI)	*P*
Continuous		NA	1.12 (1.07–1.16)	< 0.001	1.06 (1.01–1.12)	0.016	NA	NA
Dichotomy								
	Normal, < 6.1	77 (28.0%)	1 [Reference]	< 0.001	1 [Reference]	0.004	1 [Reference]	0.038
	Hyperglycemia, ≥ 6.1	198 (72.0%)	2.55 (1.91–3.40)		1.65 (1.17–2.32)		1.46 (1.02–2.09)	
Clinical threshold								
	Normal, <6.1	77 (28%)	1 [Reference]	< 0.001*	1 [Reference]	0.002*	1 [Reference]	< 0.001*
	Mild, 6.1–7.8	96 (34.9%)	2.04 (1.47–2.84)		1.50 (1.03–2.18)		1.18 (0.79–1.75)	
	Moderate, 7.8–10.0	59 (21.5%)	3.58 (2.43–5.30)		1.84 (1.14–2.98)		1.64 (0.93–2.91)	
	Severe, ≥ 10.0	43 (15.6%)	3.03 (1.98–4.64)		2.08 (1.24–3.50)		2.01 (1.05–3.88)	
Quartile								
	Q1(1.60–5.39)	39 (14.2%)	1 [Reference]	< 0.001*	1 [Reference]	0.008*	1 [Reference]	< 0.001*
	Q2(5.40–6.19)	52 (18.9%)	1.25 (0.81–1.96)		1.12 (0.68–1.85)		1.07 (0.65–1.75)	
	Q3(6.20–7.59)	72 (26.2%)	1.96 (1.28–2.98)		1.35 (0.84–2.19)		1.22 (0.70–2.13)	
	Q4(7.60–35.9)	112 (40.7%)	3.38 (2.26–5.04)		1.84 (1.13-3.00)		1.99 (1.15–3.47)	

* P for trend

NA: Not Applicable; CI: Confidence Interval; OR: Odds Ratio; PSM: Propensity Scores Matching

**Fig. 3:**
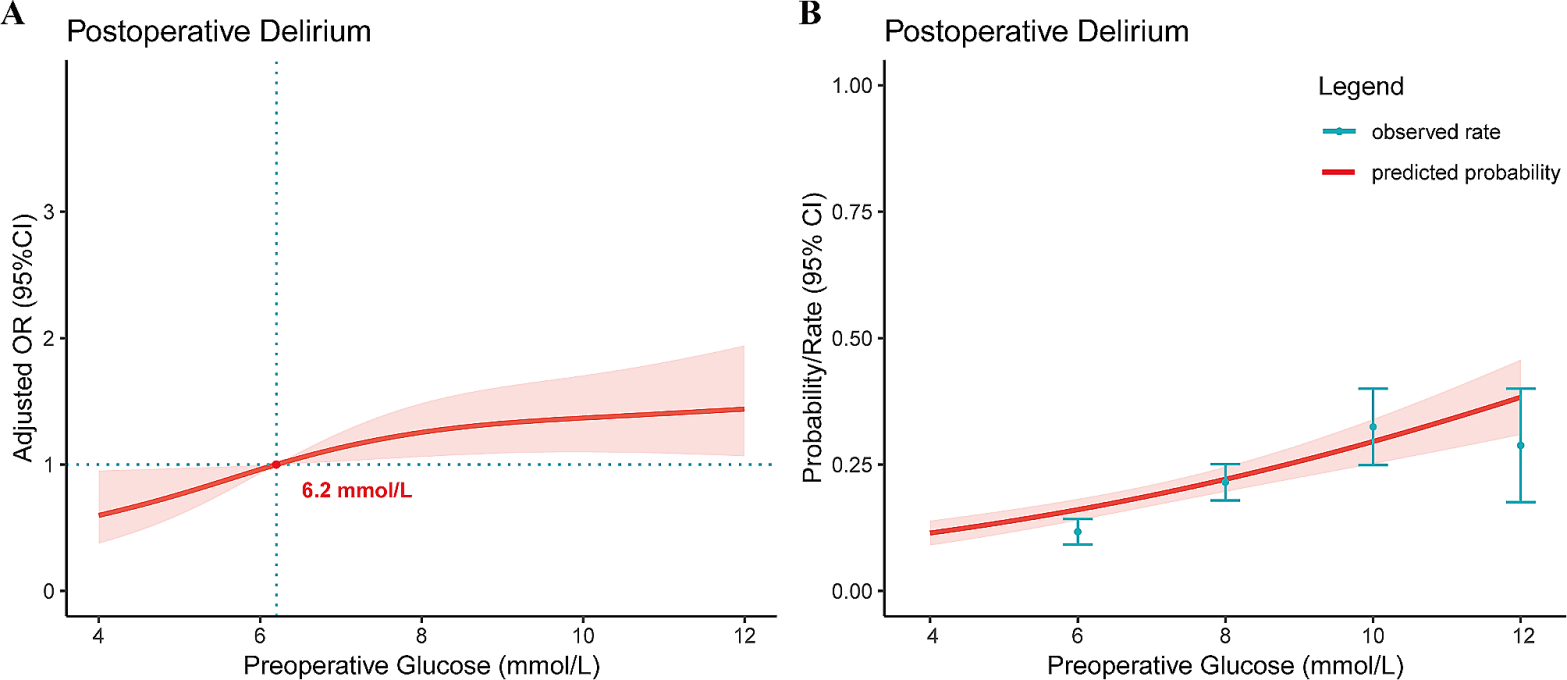
Association between preoperative glucose levels with risk (**A**) and predicted probability (**B**) of POD in patients with hip fractures. The restricted cubic spline plot (**A**) has been adjusted for all included covariates. The shaded areas represent the 95% confidence intervals

The restricted cubic spline curve (Fig. 3A) illustrates visually the relationship between preoperative glucose levels and POD in hip fracture patients. This model adjusted for all covariates included in this study. The results indicate that, as preoperative glucose levels rise, the risk of POD increases (P for Nonlinear = 0.09). Furthermore, when the preoperative blood glucose level exceeds the threshold of 6.2 mmol/L, the harmful effects of the preoperative glucose level outweigh the protective effects. Figure 3B depicts the relationship between preoperative glucose levels and the predicted probability of POD, suggesting that higher preoperative glucose levels correspond to a greater incidence rate of POD.

**Fig. 4:**
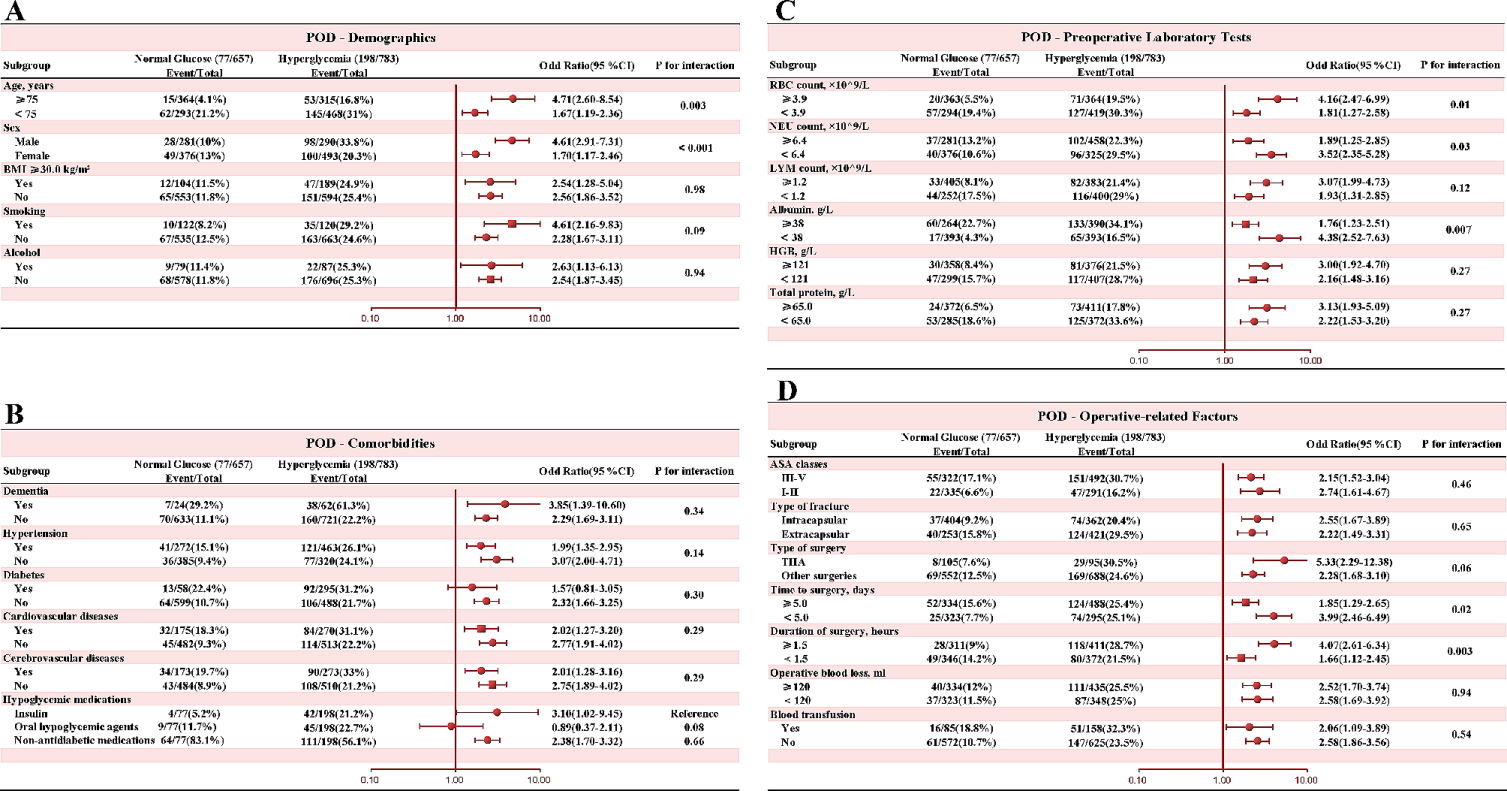
The subgroup analysis was conducted to assess potential interactions between hyperglycemia and each covariate

Subgroup analysis (Fig. 4) demonstrates that age, gender, neutrophil count, red blood cell count, albumin, time to surgery, and surgical duration exhibit interactive effects on the association between preoperative persistent hyperglycemia and POD (interaction *p* < 0.05). Specifically, among patient groups aged 75 and above, male patients, those with preoperative serum albumin levels < 38 g/L, red blood cell counts greater than 3.9 × 10^9/L, neutrophil counts less than 6.4 × 10^9/L, those undergoing surgery within 5 days of time to surgery, and those with surgery duration exceeding 1.5 h, the same preoperative blood glucose levels predict a higher risk of POD occurrence. Given the strong association between hyperglycemia and diabetes, we conducted further investigation and identified interactions between diabetes diagnosis and hyperglycemia across various hyperglycemic thresholds (Table 3). Upon raising the threshold for hyperglycemia to 7.8 mmol/L, we observed a reversal of the previously insignificant interaction between preoperative hyperglycemia and diabetes diagnosis. Our findings indicate that among patients previously diagnosed with diabetes, sustained preoperative hyperglycemia (> 7.8 mmol/L) was associated with a higher risk of POD.

**Table 3:** Diabetes-hyperglycemia interaction across varying cut-offs for hyperglycemia

Cut-off for hyperglycemia	6.1 mmol/L		7.8 mmol/L		10.0 mmol/L	
	OR (95% CI)	*p* for interaction	OR (95% CI)	*p* for interaction	OR (95% CI)	*p* for interaction
Patients with diabetes	1.57 (0.81–3.05)	0.304	2.32 (1.66–3.25)	< 0.001	1.05 (0.63–1.76)	0.148
Patients without diabetes	2.32 (1.66–3.25)		0.95 (0.60–1.50)		1.92 (1.02–3.60)	

CI: Confidence Interval; OR: Odds Ratio

## Discussion

In recent years, POD has emerged as a significant challenge for geriatric patients with traumatic hip fractures [9, 27]. However, conflicting conclusions have generated clinical uncertainty regarding the management of preoperative blood glucose levels to reduce the incidence of POD. To address these discrepancies and provide valuable insights for clinicians, our study focused on exploring the relationship between preoperative persistent hyperglycemia and POD. The findings of our study revealed a linear dose-response relationship between preoperative serum glucose levels and POD in hip fracture patients (P for Nonlinear = 0.09). Furthermore, our study identified a critical threshold value of 6.2 mmol/L, beyond which the hyperglycemia became a risk factor of POD.

The prevention and management of POD are complex due to the lack of full understanding of its underlying pathogenic mechanisms. Extensive research suggests that inadequate energy supply to the brain can lead to impaired glucose metabolism, disrupted protein synthesis, imbalanced ion levels, impaired mitochondrial function, and accelerated fat breakdown, ultimately resulting in the occurrence of POD [23].

Energy deficiency in the brain has long been considered a potential mechanism for the occurrence of POD [15]. Illuminating studies by Caplan [28] have shed light on this notion, revealing intriguing anomalies during delirium such as heightened lactate concentration within the cerebrospinal fluid, indicative of anaerobic glycolysis, and impaired glucose uptake. These findings eloquently suggest the presence of cerebral hypometabolism as a plausible causative factor behind the bewildering delirium phenomenon. Building upon Caplan’s groundwork, Titlestad et al. [15]. embarked on a groundbreaking investigation, meticulously comparing the distribution of substances associated with energy metabolism in the blood and cerebrospinal fluid of geriatric patients with hip fractures experiencing delirium. Astonishingly, their findings unveiled elevated levels of branched-chain amino acids (BCAAs) and their metabolite 3-hydroxyisobutyric acid (3-HIB), along with increased ketone body acetoacetate (AcAc) within the cerebrospinal fluid of patients with delirium, surpassing those observed in the blood serum. The presence of BCAAs and 3-HIB serves as tangible markers of insulin resistance [29, 30], while AcAc, derived from fatty acid β-oxidation, emerges as a compensatory energy source when neurons face compromised glucose utilization [31, 32]. Hence, Titlestad et al. astutely proposed that impaired glucose utilization and brain insulin resistance stand as viable mechanistic underpinnings of delirium. Wang et al. [33]. , meticulously quantifying insulin levels in the blood serum and cerebrospinal fluid of geriatric patients with hip fractures and delirium. Their findings revealed a significant reduction in cerebrospinal fluid insulin concentration among delirium patients, leading to the conclusion that preoperative insulin resistance exerts a profound influence on the occurrence of delirium. Remarkably, a plethora of studies utilizing cutting-edge FDG-PET imaging techniques have lent further support to the aforementioned discoveries by showcasing diminished glucose metabolism within the brains of patients with delirium [34–36].

Unfortunately, the brain heavily relies on glucose as its primary energy source, accounting for over 50% of total body glucose consumption [23]. Brain insulin resistance, as proposed by Arnold’s study [37], indicates that insulin receptors are expressed in various cell types in the brain, with most insulin in the cerebrospinal fluid originating from circulating pancreatic insulin. Insulin primarily enters the brain through selective transport across the blood-brain barrier, which decreases with insulin resistance or advancing age, leading to reduced insulin levels in the cerebrospinal fluid [37]. Moreover, the presence of preoperative persistent hyperglycemia in geriatric patients suggests decompensated pancreatic function, indicating inadequate insulin secretion or insulin resistance within the body [38, 39]. Conversely, the management of blood glucose levels may mitigate the progression of pancreatic function deterioration and the worsening of insulin resistance [37, 40].

In conclusion, the interaction between insulin resistance and persistent hyperglycemia disrupts brain energy supply, which forms the core of this potential mechanism. Consequently, persistent preoperative hyperglycemia may contribute to the occurrence of POD through insulin resistance and declining insulin levels. Several studies [41, 42] have also suggested that intranasal insulin administration before surgery can reduce the incidence of POD in geriatric patients, highlighting the practical significance of understanding the molecular synergistic mechanisms between insulin and serum glucose for precise prevention of POD in clinical settings. Furthermore, there are reports indicating that high blood glucose levels can directly induce neuroinflammation and promote the release of inflammatory cytokines, leading to oxidative damage and cognitive impairment [43–45]. In contrast, insulin demonstrates anti-inflammatory effects [46, 47].

### Limitations

It is crucial to disclose the limitations inherent in this study. Firstly, our reliance on preoperative glucose level assessments may limit our ability to accurately capture longitudinal fluctuations in glucose levels. Employing dynamic monitoring of glucose levels preoperatively, intraoperatively, and postoperatively could offer a more comprehensive understanding of the relationship between hyperglycemia and POD. Secondly, the retrospective design of our study introduced inherent biases, despite our efforts to mitigate them through statistical methods. Consequently, residual risks associated with these limitations persist. Lastly, although we meticulously considered numerous covariates, the potential presence of unidentified confounding factors may still impact the validity of our findings.

## Conclusions

A linear positive correlation has been observed between preoperative persistent glucose levels and the incidence of POD in patients undergoing hip fracture surgery. This association exhibited heightened significance among elderly male patients, those with concurrent hypoalbuminemia, and individuals enduring longer surgical durations. Our study identified a risk threshold for preoperative glucose levels at 6.2 mmol/L, and avoiding higher glucose levels could effectively mitigate the risk of POD occurrence. Moreover, it is imperative to underscore the importance of holistic patient health assessment and personalized glucose management strategies, as they can yield substantial health benefits beyond solely mitigating POD risks.

### Electronic supplementary material

Below is the link to the electronic supplementary material.

Supplementary Material 1

## Data Availability

All the data used and analyzed during the current study are available from the corresponding author upon reasonable request.

## Abbreviations

POD: Postoperative Delirium
PSM: Propensity Scores Matching
CAM: Confusion Assessment Method
BMI: Body Mass Index
ASA: American Society of Anesthesiologists
CI: Confidence Interval
OR: Odds Ratio
3-HIB: 3-Hydroxyisobutyric Acid
AcAc: Acetoacetate
BCAA: Branched-Chain Amino Acid
