# Supplementary material for: Association between preoperative persistent hyperglycemia and postoperative delirium in geriatric hip fracture patients
Source: BMC Geriatr. 2024 Jul 8;24:585. doi: 10.1186/s12877-024-05192-x (PMC11232206; doi:10.1186/s12877-024-05192-x)
Supplement: Supplementary file 1 — Supplementary Material 1 [file 12877_2024_5192_MOESM1_ESM.docx]

**Appendix:**

**e-Table1 Multivariate Analysis for Postoperative Delirium**

**e-Table2 Characteristics of patients before and after Propensity Score Matching based on** **preoperative glucose levels (normal < 6.1 mmol/L vs. low ≥ 6.1 mmol/L)**

**e-Table1 Multivariate Analysis for Postoperative Delirium**

| **Characteristics** | **Univariate** | | | **Multivariate** | | |
| --- | --- | --- | --- | --- | --- | --- |
|  | **OR** | **95%CI** | ***p*** | **OR** | **95%CI** | ***p*** |
| **Demographics** |  |  |  |  |  |  |
| Age, years | 1.10 | 1.08-1.11 | <0.001 | 1.08 | 1.06-1.10 | <0.001 |
| Male | 1.37 | 1.05-1.78 | 0.02 | 1.63 | 1.18-2.26 | 0.003 |
| BMI (≥30.0 kg/m²) | 1.09 | 0.79-1.50 | 0.61 | <NA> | <NA> | <NA> |
| Smoking | 0.96 | 0.68-1.37 | 0.83 | <NA> | <NA> | <NA> |
| Alcohol | 0.97 | 0.64-1.47 | 0.88 | <NA> | <NA> | <NA> |
| **Comorbidities** |  |  |  |  |  |  |
| ASA (≥III VS ＜III) | 2.74 | 2.03-3.68 | <0.001 | 1.39 | 0.98-1.97 | 0.069 |
| Dementia | 5.36 | 3.43-8.38 | <0.001 | 3.43 | 2.02-5.82 | <0.001 |
| Hypertension | 1.48 | 1.13-1.93 | <0.001 | 0.95 | 0.68-1.32 | 0.757 |
| Diabetes | 2.28 | 1.72-3.02 | <0.001 | 2.01 | 0.92-4.42 | 0.082 |
| Hypoglycemic medications | 0.64 | 0.54-0.76 | <0.001 | 0.93 | 0.58-1.5 | 0.768 |
| Cardiovascular diseases | 1.85 | 1.41-2.43 | <0.001 | 0.99 | 0.71-1.38 | 0.956 |
| Cerebrovascular diseases | 2.15 | 1.64-2.82 | <0.001 | 1.43 | 1.04-1.96 | 0.029 |
| **Operative-related Factors** |  |  |  |  |  |  |
| Type of fracture | 0.63 | 0.51-0.78 | <0.001 | 0.95 | 0.69-1.31 | 0.764 |
| Type of surgery | 0.89 | 0.80-0.99 | 0.03 | 0.93 | 0.78-1.10 | 0.4 |
| Time to surgery, days | 1.06 | 1.01-1.12 | 0.01 | 0.99 | 0.93-1.05 | 0.736 |
| Duration of surgery, hours | 1.16 | 1.01-1.35 | 0.04 | 1.25 | 0.99-1.55 | 0.054 |
| Operative blood loss, ml | 1.001 | 1.0007-1.002 | 0.03 | 1.001 | 1.00-1.002 | 0.154 |
| Blood transfusion | 1.81 | 1.32-2.49 | <0.001 | 0.86 | 0.56-1.33 | 0.503 |
| **Preoperative Laboratory Tests** |  |  |  |  |  |  |
| RBC count, ×10^9^/L | 0.52 | 0.42-0.63 | <0.001 | 0.86 | 0.52-1.43 | 0.556 |
| NEU count, ×10^9^/L | 1.05 | 1.01-1.10 | 0.04 | 1.008 | 0.95-1.07 | 0.78 |
| LYM count, ×10^9^/L | 0.59 | 0.46-0.77 | <0.001 | 0.84 | 0.66-1.07 | 0.164 |
| HGB, g/L | 0.98 | 0.97-0.99 | <0.001 | 1.0 | 0.98-1.02 | 0.979 |
| Albumin, g/L | 0.87 | 0.84-0.89 | <0.001 | 0.9 | 0.86-0.94 | <0.001 |
| Total protein, g/L | 0.93 | 0.92-0.95 | <0.001 | 0.99 | 0.97-1.03 | 0.862 |
| Blood glucose, mmol/L | 1.12 | 1.07-1.16 | <0.001 | 1.06 | 1.01-1.12 | 0.016 |

NA, Not Applicable; CI, Confidence Interval; OR, Odds Ratio; BMI, Body Mass Index; ASA, American Society of Anesthesiologists; RBC, Red Blood Cells; NEU, Neutrophils; LYM, Lymphocytes; HGB, Hemoglobin.

**e-Table2 Characteristics of patients before and after Propensity Score Matching based on** **preoperative glucose levels (normal < 6.1 mmol/L vs. low ≥ 6.1 mmol/L)**

| **Characteristics** | **Before matching** | | | **After matching** | | |
| --- | --- | --- | --- | --- | --- | --- |
|  | **Normal glucose (n=657)** | **Hyperglycemia (n=783)** | **SMD** | **Normal glucose (n=426)** | **Hyperglycemia (n=426)** | **SMD** |
| **Demographics** |  | | | | | |
| Age, years | 72.8 ± 10.3 | 76.4 ± 9.0 | 0.372 | 75.7 ± 10.0 | 75.2 ± 9.2 | 0.045 |
| Male | 281 (42.8%) | 290 37.0%) | 0.117 | 171 (40.1%) | 178 (41.8%) | 0.033 |
| BMI ≥30.0 kg/m² | 104 (15.8%) | 189 (24.1%) | 0.209 | 75 (17.6%) | 78 (18.3%) | 0.018 |
| Smoking | 122 (18.6%) | 120 (15.3%) | 0.086 | 75 (17.6%) | 75 (17.6%) | <0.001 |
| Alcohol | 79 (12.0%) | 87 (11.1%) | 0.029 | 49 (11.5%) | 55 (12.9%) | 0.043 |
| **Comorbidities** |  | | | | | |
| ASA classes ≥III | 322 (49.0%) | 492 (62.8%) | 0.281 | 249 (58.5%) | 241 (56.6%) | 0.038 |
| Dementia | 24 (3.7%) | 62 (7.9%) | 0.183 | 24 (5.6%) | 18 (4.2%) | 0.065 |
| Hypertension | 272 (41.4%) | 463 (59.1%) | 0.36 | 215 (50.5%) | 215 (50.5%) | <0.001 |
| Diabetes | 58 (8.8%) | 295 (37.7%) | 0.726 | 57 (13.4%) | 64 (15.0%) | 0.047 |
| Hypoglycemic medications |  |  |  |  |  |  |
| Insulin | 30 (4.6%) | 130 (16.6%) | 0.624 | 29 (6.8%) | 29 (6.8%) | 0.004 |
| Oral hypoglycemic agents | 28 (4.3%) | 152 (19.4%) |  | 28 (6.6%) | 29 (6.8%) |  |
| Non-antidiabetic medications | 599 (91.2%) | 501 (64.0%) |  | 369 (86.6%) | 368 (86.4%) |  |
| Cardiovascular diseases | 175 (26.6%) | 270 (34.5%) | 0.171 | 135 (31.7%) | 123 (28.9%) | 0.061 |
| Cerebrovascular diseases | 173 (26.3%) | 273 (34.9%) | 0.186 | 132 (31.0%) | 125 (29.3%) | 0.036 |
| **Operative-related Factors** |  | | | | | |
| Type of fracture |  |  |  |  |  |  |
| Femoral neck fracture | 404 (61.5%) | 362 (46.2%) | 0.291 | 224 (52.6%) | 221 (51.9%) | 0.008 |
| Intertrochanteric fracture | 220 (33.5%) | 363 (46.4%) |  | 176 (41.3%) | 180 (42.3%) |  |
| Subtrochanteric fracture | 33 (5.0%) | 58 (7.4%) |  | 26 (6.1%) | 25 (5.9%) |  |
| Type of surgery |  |  |  |  |  |  |
| Total Hip Arthroplasty | 105 (16.0%) | 95 (12.1%) | 0.152 | 59 (13.8%) | 61 (14.3%) | 0.008 |
| Hemiarthroplasty | 156 (23.7%) | 200 (25.5%) |  | 119 (27.9%) | 109 (25.6%) |  |
| Intramedullary nail fixation | 175 (26.6%) | 305 (39.0%) |  | 140 (32.9%) | 144 (33.8%) |  |
| Fixation with steel plate | 65 (9.9%) | 112 (14.3%) |  | 48 (11.3%) | 58 (13.6%) |  |
| Fixation with hollow nails | 156 (23.7%) | 71 (9.1%) |  | 60 (14.1%) | 54 (12.7%) |  |
| Time to surgery, days | 5.1 ± 2.6 | 5.7 ± 2.6 | 0.242 | 5.4 ± 2.6 | 5.4 ± 2.6 | 0.018 |
| Duration of surgery, hours | 1.6 ± 0.7 | 1.7 ± 0.8 | 0.15 | 1.7 ± 0.8 | 1.6 ± 0.8 | 0.03 |
| Operative blood loss, ml | 160.5 ± 148.7 | 183.4 ± 161.7 | 0.147 | 173.3 ± 162.5 | 165.0 ± 117.6 | 0.059 |
| Blood transfusion | 85 (12.9%) | 158 (20.2%) | 0.196 | (16.2%) | (16.0%) | 0.006 |
| **Preoperative Laboratory Tests** |  | | | | | |
| RBC count, ×10^9^/L | 4.0 ± 0.7 | 3.9 ± 0.7 | 0.199 | 3.9 ± 0.6 | 3.9 ± 0.7 | 0.013 |
| NEU count, ×10^9^/L | 6.1 ± 2.5 | 7.3 ± 2.8 | 0.423 | 6.8 ± 2.5 | 6.7 ± 2.3 | 0.043 |
| LYM count, ×10^9^/L | 1.4 ± 0.7 | 1.2 ± 0.6 | 0.26 | 1.3 ± 0.5 | 1.3 ± 0.7 | 0.019 |
| HGB, g/L | 121.6 ± 20.4 | 118.2 ± 20.7 | 0.168 | 118.9 ± 20.4 | 119.0 ± 21.2 | 0.009 |
| Albumin, g/L | 38.2 ± 5.0 | 37.1 ± 5.2 | 0.212 | 37.3 ± 4.9 | 37.6 ± 5.1 | 0.058 |
| Total protein, g/L | 65.3 ± 6.9 | 64.7 ± 7.8 | 0.087 | 64.7 ± 6.6 | 65.0 ± 7.4 | 0.046 |

Continuous variables are presented as mean ± standard deviation, while categorical variables are represented by numbers (percentages).

SMD, Standardized Mean Difference; CI, Confidence Interval; OR, Odds Ratio; BMI, Body Mass Index; ASA, American Society of Anesthesiologists; RBC, Red Blood Cells; NEU, Neutrophils; LYM, Lymphocytes; HGB, Hemoglobin.
